# Resonance-Amplified Terahertz Near-Field Spectroscopy of a Single Nanowire

**DOI:** 10.1021/acs.nanolett.4c04395

**Published:** 2024-11-26

**Authors:** Sarah Norman, Greg Chu, Kun Peng, James Seddon, Lucy L Hale, Hark Hoe Tan, Chennupati Jagadish, Ralf Mouthaan, Jack Alexander-Webber, Hannah J Joyce, Michael B Johnston, Oleg Mitrofanov, Thomas Siday

**Affiliations:** † Electronic and Electrical Engineering, University College London, London WC1E 7JE, United Kingdom; ‡ Department of Engineering, University of Cambridge, Cambridge CB3 0FA, United Kingdom; § Department of Physics, University of Oxford, Clarendon Laboratory, Parks Road, Oxford OX1 3PU, United Kingdom; ∥ Institute of Quantum Electronics, ETH Zurich, Auguste-Piccard-Hof 1, 8093 Zürich, Switzerland; ⊥ Australian Research Council Centre of Excellence for Transformative Meta-Optical Systems, Department of Electronic Materials Engineering, Research School of Physics, The Australian National University, Canberra, Australian Capital Territory 2600, Australia; # Centre of Light for Life, University of Adelaide, North Terrace, Adelaide, South Australia 5005, Australia; 7 School of Physics and Astronomy, University of Birmingham, Birmingham B15 2TT, United Kingdom

**Keywords:** Near-field microscopy, Ultrafast dynamics, Terahertz spectroscopy, Nanowires

## Abstract

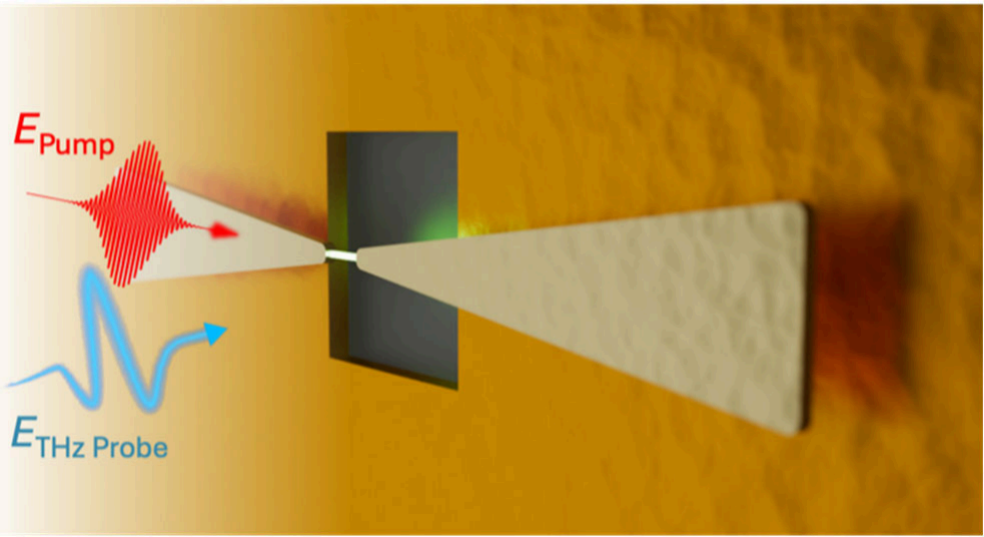

Nanoscale material systems are central to next-generation optoelectronic and quantum technologies, yet their development remains hindered by limited characterization tools, particularly at terahertz (THz) frequencies. Far-field THz spectroscopy techniques lack the sensitivity for investigating individual nanoscale systems, whereas in near-field THz nanoscopy, surface states, disorder, and sample-tip interactions often mask the response of the entire nanoscale system. Here, we present a THz resonance-amplified near-field spectroscopy technique that can detect subtle conductivity changes in isolated nanoscale systems—such as a single InAs nanowire—under ultrafast photoexcitation. By exploiting the spatial localization and resonant field enhancement in the gap of a bowtie antenna, our approach enables precise measurements of the nanostructures through shifts in the antenna resonant frequency, offering a direct means of extracting the system response, and unlocking investigations of ultrafast charge-carrier dynamics in isolated nanoscale and microscale systems.

Revealing the fundamental characteristics which underpin the optical and electronic response of material systems with micro- and nanoscale geometry is essential for the development of future communication and computing technologies. Terahertz (THz) spectroscopy has become a powerful approach for noncontact material characterization, providing direct access to collective charge, spin and lattice excitations as well as light-induced dynamics over ultrafast time scales.^1,2^ For practical semiconductor devices, it also enables probing of key physical processes and critical parameters.^3−7^ Yet, the spatial resolution of conventional THz techniques is limited by diffraction to hundreds of microns. This makes measuring individual nano- and microscale systems–such as nanowires–near impossible.^8,9^ Instead, these systems are measured as ensembles numbering hundreds or thousands. While these measurements have proven effective in extracting ensemble-averaged material quantities such as mobility,^4^ inhomogeneous broadening masks the response of individual nanoscale elements.^1^

Near-field techniques overcome the diffraction limit, enabling investigations of individual nanostructures across a range of length scales, from microscopic^10−13^ to nanoscopic^14−19^ and even atomic.^20,21^ These approaches have been used to explore a variety of physical processes, from charge accumulation at the nanoscale^22,23^ to the controlled launch of THz surface plasmons.^12,24−26^ However, methods which resolve down to the nanoscale–despite their potential for deeply subwavelength spatial resolution–struggle to measure the entire interaction between light and matter in nanoscale systems. This is because nanoscopy is heavily influenced by surface defect states,^27^ disorder^14^ and other phenomena which naturally emerge over nanometer length scales.^28^ These can dominate over the aggregate response of nanostructured systems. As a result, while these techniques excel at investigating fundamental phenomena at extreme length scales, they often face challenges in accessing the response of the entire nano- and microscale systems.

Here, we demonstrate an alternative approach to probing nanoscale systems by coupling them to a resonant THz antenna excited by single-cycle THz pulses. Through external modulation, changes in the properties of the nanoscale system–in our case a single InAs nanowire–will induce subtle changes in the antenna’s resonant frequency, which we detect by capturing the evanescent field of the antenna using aperture-type THz near-field spectroscopy. We demonstrate high sensitivity of this resonance-amplified near-field spectroscopy approach by photoexciting the nanowire with a femtosecond optical pulse and detecting a small plasma frequency increase of Δν∼1 THz, equivalent to adding ∼10^2^ electrons. Furthermore, the changes in the antenna response allow us to quantitatively sample the carrier density in a single nanowire under ultrafast photoexcitation without requiring analytical approximations.^29^ This analysis is enabled by full-wave numerical simulations of the entire antenna-nanowire-probe configuration. It provides a direct method for extracting the system response, unlocking investigations of ultrafast excitations and charge carrier dynamics in single nanoscale and microscale systems.

For systems significantly smaller than the wavelength of the incident THz radiation, the coupling efficiency of THz energy from a freely propagating beam into the system is very low.^8^ To enhance the sensitivity of our approach, we use the spatial localization and resonant field enhancement within the gap of a gold bowtie antenna. Near the resonant frequency of the antenna, the field strength in the gap is enhanced by a factor of ∼50 (gap size 5 μm) and localized to within a micron-scale volume (Figure 1a,b). This field enhancement means that the gap region of the antenna is highly sensitive to local changes in conductivity,^30^ which can be detected with a near-field probe (Figure 1c) by observing changes in the antenna’s resonant frequency or amplitude. We used a custom-made aperture-type near-field probe sensitive to THz plasmonic excitations.^8,26,31^ This allows us to detect the evanescent fields on the bowtie antenna and enables spectroscopic analysis.

**Figure 1:**
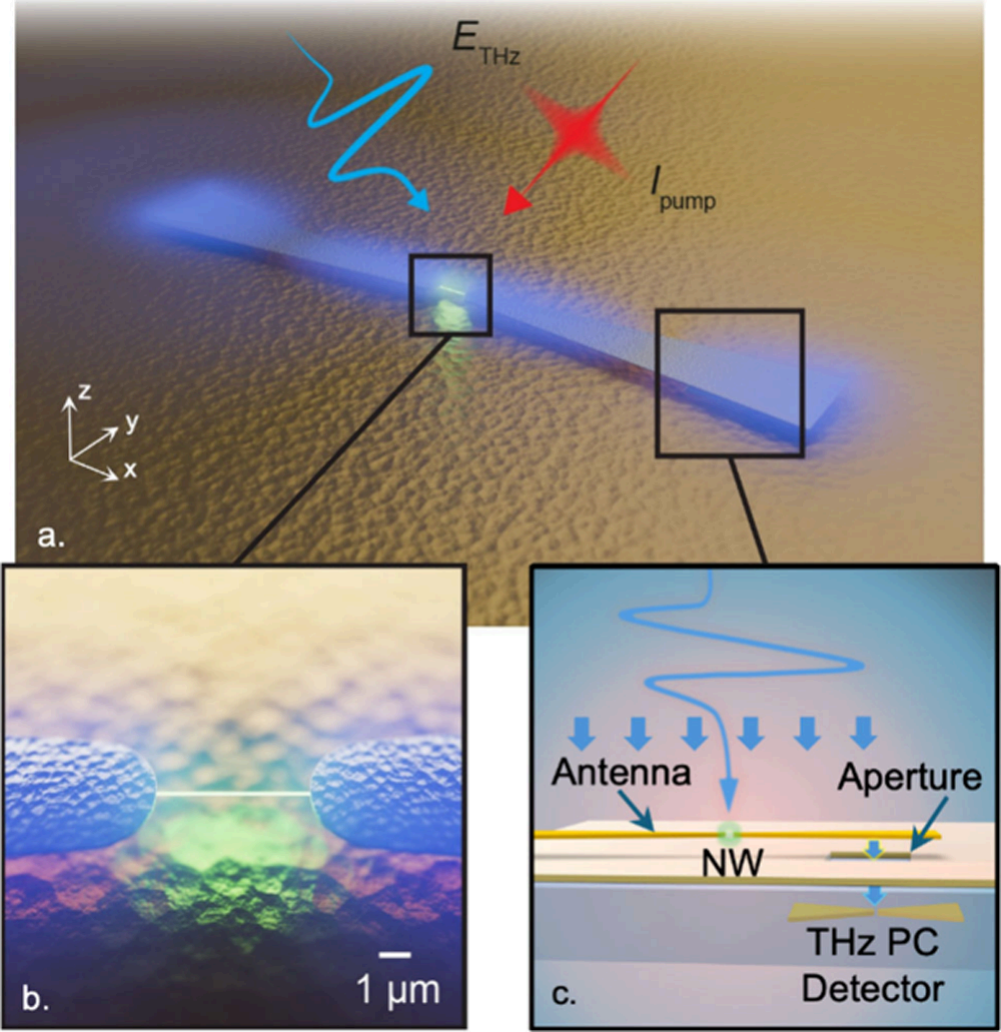
(a) Conceptual illustration of resonance-amplified THz near-field spectroscopy. The aperture probe is positioned behind the right side of a metallic bowtie antenna which resonantly enhances and spatially localizes an incident THz pulse (blue), enabling detection of small changes in THz conductivity of a single InAs nanowire (b) positioned between the antenna tips. Conductivity changes in the nanowire induced by the optical pump (red) result in small shifts in the antenna’s resonant frequency. Antenna fields (shown as blue glow)—which encode information about the nanowire conductivity—are coupled through the subwavelength aperture probe (c), where they are directly detected with an integrated THz photoconductive detector.

To illustrate our concept, we first consider an antenna with an open gap. We excite the antenna with a THz pulse incident from the substrate side (Figure 1a and Supporting Information, section 1) and measure the evolution of the antenna’s near-field signal, *E*(*t*). We see a resonant response appearing as oscillations in the time-domain waveforms which persist for several cycles (Figure 2a), resulting in a peak in the signal spectrum at a frequency of ν_R_ = 1.4 THz (solid lines, Figure 2b). This open antenna signature serves as an example of the ideal response without any conductive material in the gap. In contrast, for a highly conductive sample, we introduce a small 2 μm wide metallic bar in the gap of the bowtie, shorting the antenna. Here, we observe a dramatic reduction in the resonant frequency down to 0.6 THz. This shift arises from the fundamental dipolar resonance: in the open antenna case, the bowtie antenna acts as two separate—yet still coupled—resonators, each with a fundamental dipolar resonance at ν_R_. For the shorted antenna, it behaves as a single resonator with a fundamental dipolar resonance at ∼ν_R_/2. We confirmed the dipolar nature of the two resonances by raster-scanning the antenna relative to the aperture and spatially mapping the near-field signal representing the THz surface currents in the antenna^10^ at a peak in the time-domain waveform (Figure 2e). This clear frequency shift between the two antenna configurations demonstrates how the bowtie antenna’s field enhancement and the aperture probe allow us to detect conductivity changes in a nanoscale sample placed within the antenna gap.

**Figure 2:**
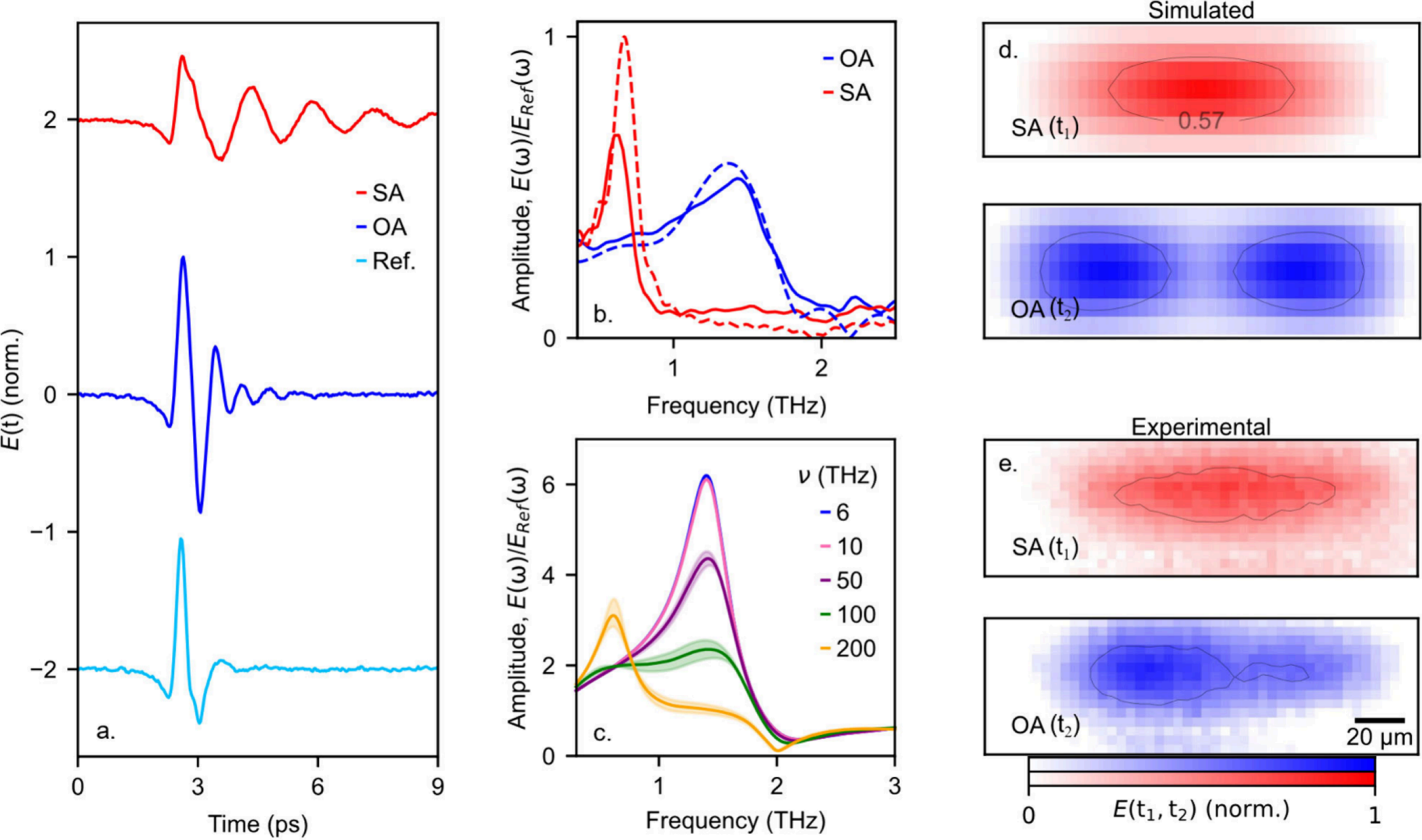
(a) Experimentally measured time-domain THz near-field signal, *E*(*t*), recorded by the aperture probe when positioned near the center of the bowtie antenna for two cases: an open antenna (OA, blue) and shorted antenna (SA, red), as well as a reference signal acquired away from the antenna on the sample substrate (light blue). (b) Experimentally measured (solid line) and simulated (dashed line) spectral amplitude for OA and SA normalized to the reference spectrum, showing a dramatic shift in resonant frequency. (c) Simulated spectral amplitude sampled by the aperture probe for a material with increasing THz conductivity, defined by the Drude model with the plasma frequency (ν) ranging from 6–200 THz. The background carrier density and scattering rate are obtained from previous far-field studies.^4^ Shaded regions show the variation of the system response due to the uncertainty in the measured scattering rates. (d) Simulated and (e) measured near-field signal maps obtained with the aperture probe captured at peaks in the time-domain waveforms (*t*_1_ = 4.3 ps, *t*_2_ = 3.4 ps). Here the isoline data are smoothed using a Savitzky–Golay filter with a four-point window. The bowtie antennas are 69 μm in length, featuring a 5 μm gap (for the open antenna) or a 2 μm wide bar (for the shorted antenna), and fabricated on a 0.5 mm thick GaAs substrate.

To quantitatively evaluate conductivity within the antenna gap, we modeled the entire sample–antenna–probe configuration using a full-wave electromagnetic solver (CST Microwave Studio). By including the near-field probe with an integrated THz detector in the simulation (see Supporting Information, section 2), we can model the near-field signal detected by the aperture probe, allowing us to calculate its exact relation to the conductivity of the sample in the antenna gap. In Figure 2, we display normalized spectra of the simulated THz near-field signal (dashed lines, Figure 2b). The simulated spectra show excellent agreement with the experimental results, while the simulated spatial maps of the near-field signal (Figure 2d) confirm that the observed near-field images represent different antenna resonances. This demonstrates that for the two ideal cases, with extreme conductivity values, our resonance-amplified near-field spectroscopy approach can sense the response of the entire system in the gap.

Having established the framework, we now apply it to a prototypical material system: a single InAs nanowire. The charge-carrier mobility and concentration are key parameters of these nanowires; essential for assessing their potential as active components in solar cells,^32^ photodetectors^33−35^ and polarization modulators.^36^ However, measuring the response of individual nanowires as an entire system has so far been beyond the scope of current near- and far-field techniques. To assess if small changes in nanowire conductivity can be measured with our approach, we model a single InAs nanowire within the antenna gap and modulate its THz conductivity by increasing the plasma frequency, ν, from 6–200 THz. We find that the increasing conductivity of the nanowire can significantly shift the antenna response as measured by the aperture probe (Figure 2c). As the THz conductivity increases, the resonant frequency shifts from that of the open antenna signature to lower frequencies, similar to the shorted antenna. Even for small variations in the THz conductivity, corresponding to the addition of ∼10^2^ electrons in the nanowire, our approach is expected to detect changes in the antenna’s resonance, albeit subtle, revealing the collective response of nanowire charge carriers that would otherwise be beyond the sensitivity of traditional far-field THz spectroscopy techniques.

We fabricated antenna devices with single InAs nanowires in the gap and sampled their THz response, comparing them to two open antennas (Figure 3). The Fourier analysis reveals both types of antennas are characterized by a similar resonant frequency (∼1 THz) and line width (Figure 3b), consistent with low nanowire background doping^4^ and the open antenna gap. Interestingly, there is a slight variation in the response of the antenna with nanowires compared to the open antennas particularly noticeable in Nanowire 1 (NW1), which exhibits a 70 GHz spectral down-shift. This shift could indicate different background doping densities between the nanowires. However, small variations in the antenna geometry within fabrication tolerance may also play a role, making it difficult to ensure the frequency shift originates solely from changes in the nanowire conductivity.

**Figure 3:**
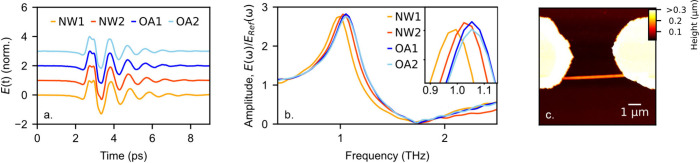
(a) Time-domain near-field signal, *E*(t), measured in the center of two antennas with nanowires (NW1, NW2) and two open antennas (OA1, OA2). (b) Corresponding amplitude spectra normalized to the reference spectrum (see Figure 2a) showing the small spectral shift between the NW1/NW2 and OA1/OA2. (c) AFM image of NW2 between the resonant antenna tips. The bowtie antenna is 129 μm in length, featuring a 5 μm gap (for the open antennas) or a single 145 nm diameter nanowire (for the antennas with nanowires), and are fabricated on a 0.5 mm thick quartz substrate.

To investigate more directly the changes in nanowire conductivity, we modulate the electron–hole pair density within a single nanowire. We photoexcite the nanowire with an above-bandgap optical pump pulse (1.55 eV, 100 fs) and probe the variations in the antenna’s resonance for a fixed delay between the optical and THz pulse of approximately 0.5 ns and fluence of 20 μJ/cm^2^ (see Supporting Information, section 1). Despite this significant delay between photoexcitation and THz probing in our experiment, the lifetime of this class of nanowires is comparable to our delay time (∼500 ps),^4^ and simulations (Figure 2c) predict we can still measure photoexcited changes to the charge-carrier density in the nanowire owing to the high sensitivity of our resonance-amplified approach.

We recorded the THz near-field signal of the antenna, *E*(*t*), by the aperture probe (without optical excitation) as illustrated in Figure 4, as well as the change induced by the optical pump pulse, Δ*E*(*t*). In these measurements, Δ*E*(*t*) represents changes in the antenna’s resonance. The near-field measurements of Δ*E*(*t*) and *E*(*t*) are shown in Figure 4a: the amplitude of the pump-induced change Δ*E*(*t*) is smaller than that of *E*(*t*) by 2 orders of magnitude and, while they show similar waveforms, the polarity of Δ*E*(*t*) is reversed, indicating that the amplitude of *E*(*t*) is reduced due to photoexcitation, consistent with the effect of increased carrier density in the nanowire.

**Figure 4:**
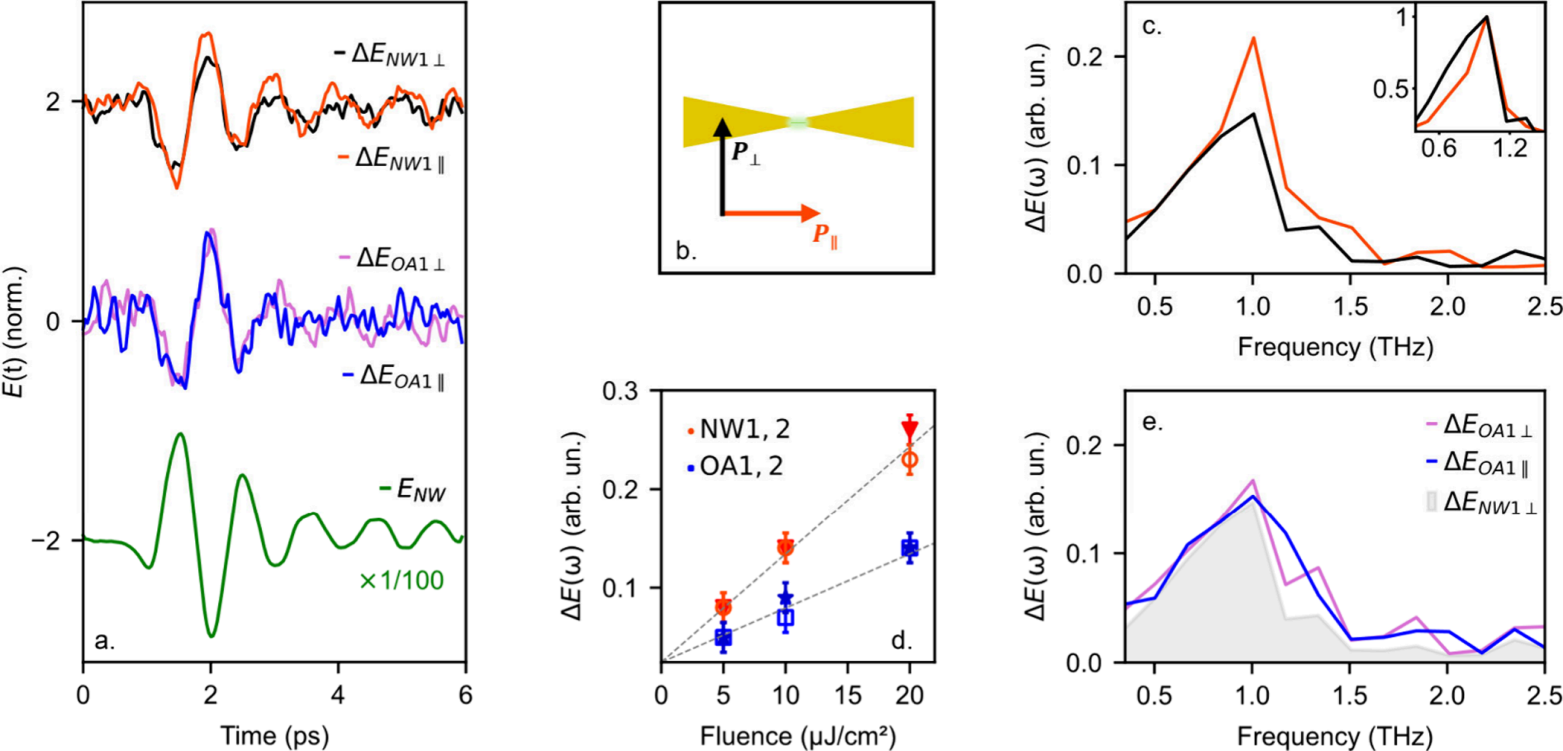
(a) Time-domain near-field signal of an antenna with nanowire without photoexcitation, *E*(t), and the corresponding photoinduced change in transmission, Δ*E*(*t*), with an optical pump polarized perpendicular, *P*_⊥_, and parallel, *P*_∥_, to the nanowire length, at a fixed pump–probe delay time of approximately 0.5 ns. The blue and pink traces show Δ*E*(*t*), for the open antenna for comparison. (b) Schematic of incident pump pulse for both parallel and perpendicular polarization. (c) Amplitude spectra of Δ*E*_NW2∥_ (red) and Δ*E*_NW2⊥_ (black). Inset: normalized amplitude spectra demonstrating the narrowing of resonance for parallel photoexcitation of the nanowire. (d) Peak amplitude of the spectra as functions of fluence for the two antennas with nanowires and two open antennas. (e) Amplitude spectra for the open antenna, with shaded region showing Δ*E*_NW2⊥_ provided for reference.

We also observe a similar effect, albeit smaller, in a reference open antenna, indicating that the pump also affects the near-field probe. As the wavelength of the optical pulse is centered at 800 nm, it can excite charge carriers within the aperture region of the probe, where semiconducting GaAs is present to improve THz coupling.^37^ These charge carriers slightly screen the THz field coupling through the aperture into the probe. As a result, *E*(*t*) is reduced in amplitude even in the case of the open antenna. To differentiate and isolate the nanowire response from the probe under optical excitation, we employed two techniques. First, we varied the polarization of the optical pump pulse: the incident field polarization along the nanowire is known to generate approximately 1 order of magnitude more charge carriers in the nanowire compared to the orthogonal polarization,^1^ whereas both polarizations induce the same THz pulse attenuation within the aperture (Figure 4). Second, we minimized the effect of the pump light on the aperture by positioning the aperture behind the center of one arm of the antenna (Figure 1c). This suppresses the intensity of pump light reaching the aperture while still allowing near-field detection of the THz antenna resonance (Figure 2). Together, these effectively isolate changes in the nanowire conductivity from the pump effect on the probe.

Figure 4 demonstrates the impact of changing the polarization of the optical pump pulse: when the polarization is parallel to the long axis of the nanowire, *P*_∥_, (Figure 4b), Δ*E*(ω) exhibits greater amplitude and narrower spectral shape compared to both the orthogonal pump polarization, *P*_⊥_, as well as the open antenna for both pump polarizations, suggesting that these effects result from the enhanced conductivity of the nanowire. We find this result consistently across multiple devices and excitation fluences (Figure 4d). The broader, lower amplitude response observed with the perpendicular polarization and open antennas is therefore attributed to the optical pump pulse’s effect on the probe.

For the antenna with nanowire and parallel optical pump polarization, *P*_∥_, we observe a spectral narrowing and more pronounced ringing in the time-domain waveform Δ*E*(*t*) (red waveform in Figure 4a) compared to the unpumped antenna response *E*(*t*). In contrast, for the perpendicular polarization, *P*_⊥_, we observe only a uniform spectral change, and no spectral narrowing occurs. To understand these seemingly counterintuitive results, we note that at the start of the near-field waveform *E*(*t*), the signal is dominated by the incident single-cycle THz pulse (∼0–2 ps) whereas at later times, the signal comes predominantly from current oscillations in the bowtie antenna (∼2–6 ps). The increased conductivity of the nanowire does not affect the earlier part of the waveform significantly (the incident THz pulse); however it can damp the antenna resonance, thereby reducing the strength of oscillations in the antenna. The differential Δ*E*(*t*) signal is ideal for detecting this effect, since it shows the pump-induced changes in *E*(*t*). Even small damping in the antenna resonance would appear as increased ringing in Δ*E*(*t*) with an opposite phase in comparison to *E*(*t*) and, as a result, spectral narrowing. Therefore, the stronger oscillations observed after the incident THz pulse in Δ*E*(*t*) (Figure 4a) serve as a key signature of the nanowire effect on the antenna response and indicate a modification of the antenna resonance (Figure 4c). Consequently, our approach is highly sensitive to any factors affecting the antenna’s response and enables measurements of the nanowire conductivity through subtle changes in the amplitude or frequency of the resonance.

When compared with our numerical simulations, these results allow us to extract directly the nanoscale material properties of the nanowire. Using the Drude model, we simulated a range of nanowire conductivities by varying the plasma frequency around a baseline ν = 6 THz, corresponding to the background charge-carrier density. The background carrier density and scattering rate are obtained from previous far-field studies.^4^ By modeling the changes in the THz electric field, Δ*E*(t), for different plasma frequencies, we can replicate the experimentally measured pump-induced change in the THz electric field, Δ*E*(ω)/*E*(ω). Figure 5a shows the simulated time-domain waveforms of the THz electric field detected by the probe, *E*(*t*), without optical excitation, and the small change in the THz field, Δ*E*(*t*), with optical excitation. As in the experiment, the simulations show pronounced oscillations in Δ*E*(*t*) after the incident THz pulse corresponding to the damping of the antenna’s resonance. Yet, this effect is even stronger here than in the experiment since there is no parasitic detector response in our simulations. This unequivocally demonstrates how the enhanced oscillations and spectral narrowing—observed for the nanowire with *P*_∥_—is a feature that can only originate from the nanowire itself. The Fourier analysis, overlaid with the experimental data is shown in Figure 5b. By subtracting the response for the perpendicular optical pump polarization from that for the parallel polarization, we can isolate the nanowire’s contribution to the signal and find the photoexcited carrier density in the nanowire.

**Figure 5:**
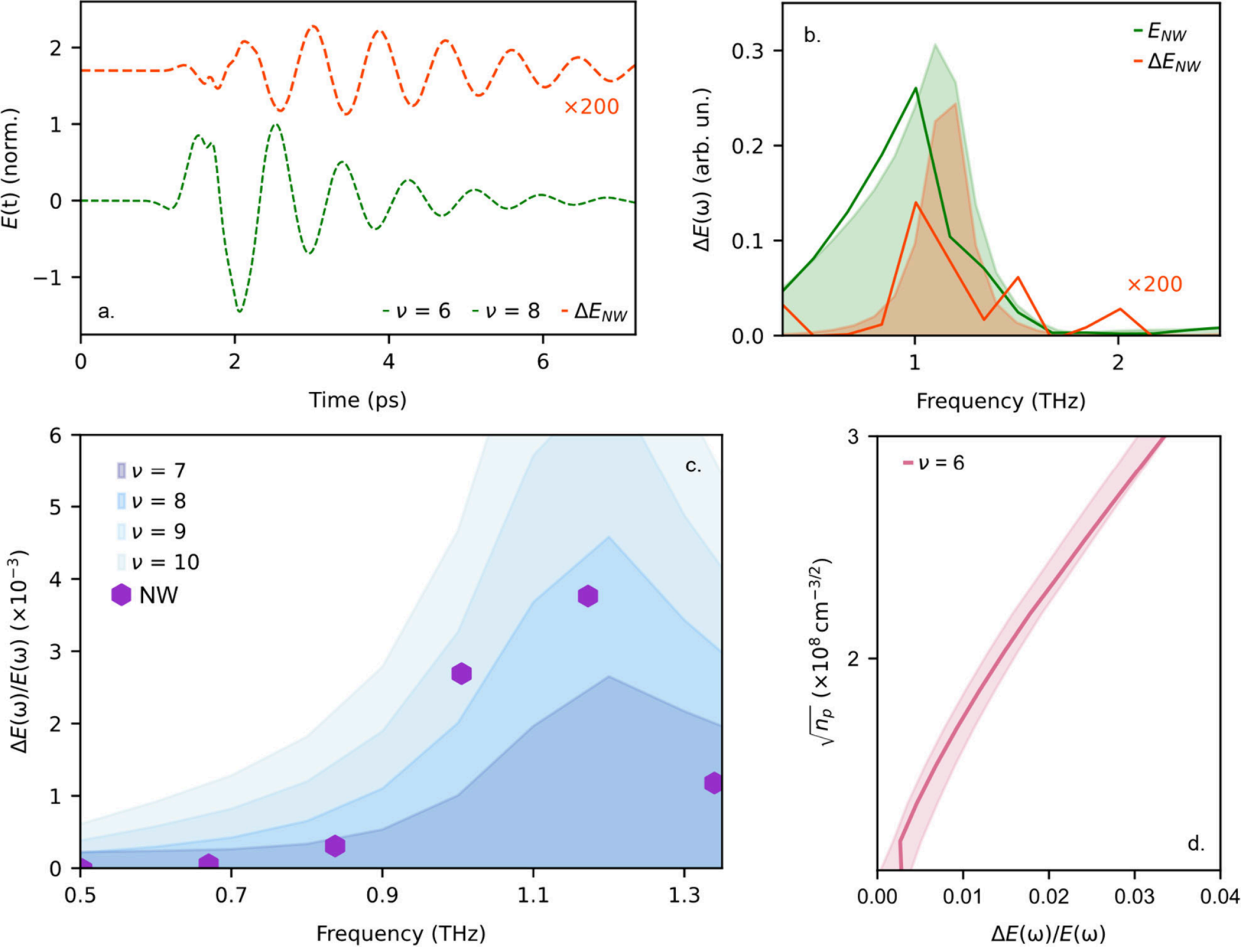
(a) Simulated time-domain waveform of near-field THz signal, *E*(*t*), of an antenna with nanowire without photoexcitation, with a background charge-carrier density corresponding to a plasma frequency, ν = 6 THz (green), and photoinduced change in THz transmission, Δ*E*(*t*), for an increasing nanowire conductivity corresponding to a plasma frequency of ν = 8 THz (orange). (b) Fourier spectral amplitude of the simulated (shaded area) and measured (solid lines) near-field THz signal, and photoinduced change in THz transmission. (c) Measured (purple markers) and simulated (blue shaded area) change in the response of the antenna with a single photoexcited nanowire in the gap, Δ*E*(ω)/*E*(ω), showing the plasma frequency in the nanowire increasing from ∼6THz to ∼8 THz. (d) Simulated pump-induced change in the near-field signal, Δ*E*(ω)/*E*(ω), for increasing carrier densities, .

The extracted photoinjected electron density was *n*_p_ = (1.8 ± 0.4) × 10^16^ cm^–3^, which corresponds to only ∼10^2^ additional electrons in the nanowire volume. As illustrated in Figure 5c, the change in the antenna’s response, Δ*E*(ω)/*E*(ω), shows the plasma frequency in the nanowire increasing from ∼6 THz to ∼8 THz, which is consistent with findings from far-field ensemble studies.^38,39^ Finally, using the simulation model, we correlated the square root of the photoexcited charge-carrier density, , with the amplitude of the pump-induced change in the THz electric field, Δ*E*(ω)/*E*(ω), (Figure 5d), providing us with a direct tool for evaluating the photoexcited charge-carrier densities.

Our method of combining resonance-amplified THz near-field spectroscopy with quantitative numerical simulations allows us to extract the nanoscale material properties of a single semiconductor nanowire through changes in the antenna’s resonance. As a proof-of principle, we focused on sampling the charge-carrier density at a fixed time-delay of approximately 0.5 ns after photoexcitation, yet the technique opens the doors to studying the ultrafast evolution of conductivity in individual nanowires and extracting a host of material properties including charge-carrier lifetimes and scattering rates. With the developed ability to model the THz near-field signal modulated by an optical pump pulse, this approach can also provide a direct means to extracting electron mobility. Lastly, we note that this technique exploits the subtle changes in the antenna’s resonance to probe the nanoscale material properties, as demonstrated here with a bowtie design which is sensitive to small increases in plasma frequency (∼1 THz). By refining the resonator design, through improved field coupling and higher Q-factor antennas, more sensitive and precise measurements of conductivity variations in micro- and nanoscale systems can be achieved.

In conclusion, this work highlights the unique potential of near-field spectroscopy, combined with the spatial localization and resonant field enhancement of a bowtie antenna, to investigate the entire response of a single InAs nanowire. With photoexcitation of the nanowire, our resonance-amplified approach probes the photoinduced charge carrier dynamics in the nanowire, free from the direct influence of surface states or disorder and enables quantitative spectroscopic analysis without relying on analytical models. The high sensitivity to subtle conductivity changes within a nanoscale system–as shown in this prototypical InAs nanowire–alongside the potential for the optical pump with variable time delay, further highlights the potential of resonance-amplified near-field spectroscopy for exploring fundamental quantum dynamics in a range of 2D materials,^40^ investigations of strong light-matter coupling^41^ or even strong-field control over light-matter interaction,^42^ all of which have proven extremely difficult to access in inherently disordered nanoscale material systems.

## Methods

### Fabrication

Wurtzite InAs nanowires (average diameter 145 ± 10 nm, grown by metal–organic chemical vapor deposition^43^) were coated with Al_2_O_3_ (∼10 nm) and dispersed on z-cut quartz substrates. The nanowire ends were etched to remove the Al_2_O_3_ coating and the native oxide. THz antennas were lithographically deposited (Ti/Au:5 nm/150 nm) over individual nanowires, as outlined in ref.^8,10^

### Resonance-Amplified THz Near-Field Spectroscopy

Each antenna was illuminated (normal incidence) with THz pulses using a THz time-domain spectroscopy setup (pumped by a Ti:sapphire laser: 800 nm, 76 MHz, ∼ 100 fs).^31^ The custom-made near-field probe with a 10 × 10 μm^2^ aperture and an integrated photoconductive antenna THz detector^10^ was positioned at ∼12 ± 2 μm from the antenna to detect and map THz currents.^10^ An optical pump beam from the Ti:sapphire laser illuminated the sample ∼0.5 ns before the THz pulses with a fluence of 20 μJ/cm^2^ (∼50 μm diameter spot, 45° incidence angle).

## Supporting Information Available

Additional information detailing the experimental setup, numerical simulations, and further fabrication details (PDF)
