# Supplementary material for: Resonance-Amplified Terahertz Near-Field Spectroscopy of a Single Nanowire
Source: Nano Lett. 2024 Nov 26;24(49):15716–23. doi: 10.1021/acs.nanolett.4c04395 (PMC11638953; doi:10.1021/acs.nanolett.4c04395)
Supplement: Supplementary file 1 — nl4c04395_si_001.pdf [file nl4c04395_si_001.pdf]

Supporting Information

Resonance-amplified terahertz near-field spectroscopy of a single nanowire

Sarah Norman[1,], Greg Chu[2], Kun Peng[3], James Seddon[1], Lucy L Hale[4], Hark Hoe Tan[5], Chennupati Jagadish[5], Ralf Mouthaan[2,6], Jack Alexander-Webber[2], Hannah J Joyce[2], Michael B Johnston[3], Oleg Mitrofanov[1,*], Thomas Siday[7]*

[1]Electronic and Electrical Engineering, University College London, London, WC1E 7JE, United Kingdom

[2]Department of Engineering, University of Cambridge, Cambridge, CB3 0FA, United Kingdom

[3]Department of Physics, University of Oxford, Clarendon Laboratory, Parks Road, Oxford, OX1 3PU, United Kingdom

[4]Institute of Quantum Electronics, ETH Zurich, Zürich, Auguste-Piccard-Hof 1, 8093 Zürich, Switzerland

[5]Australian Research Council Centre of Excellence for Transformative Meta-Optical Systems, Department of Electronic Materials Engineering, Research School of Physics, The Australian National University, Canberra, Australian Capital Territory 2600, Australia

[6]Centre of Light for Life, University of Adelaide, North Terrace, Adelaide SA 5005, Australia

[7]School of Physics and Astronomy, University of Birmingham, Birmingham, B15 2TT, United Kingdom

1. Resonance-Amplified THz Near-Field Spectroscopy

Broadband THz pulses were generated from a 1 mm thick layer of InAs in a THz time-domain spectroscopy (THz-TDS) setup pumped by 100 fs, 76 MHz pulsed light from a Ti:Sapphire oscillator laser at a ~45-degree incidence angle (Fig. S1a) [1,2]. Individual bowtie antennas were illuminated with the THz pulses at normal incidence from the substrate side (Fig. S1b). The THz pulse waveforms and spatial maps were recorded in the time-domain with a sampling interval of 0.033 ps (time-delay step of 5 μm) using an aperture-type THz near-field probe. The incident THz pulse waveform detected by the near-field probe is shown in Fig. S1c. The THz spectra were then obtained by applying a Fourier transform (Fig. S1d).

The near-field probe features a gold planar surface with a 10×10 μm^2 aperture and an integrated photoconductive antenna THz detector; the probe was positioned at ~12 ± 2μm from the sample. The probe detects the THz fields coupled through the aperture and is sensitive to the temporal derivative of the electric field parallel to the aperture plane (along the x-axis), $\frac{dE_x}{dt}$; and the spatial derivative of the out-of-plane field component, $\frac{dE_z}{dx}$ [2,3]. The latter contribution tends to dominate the near-field signal, *E(t)*, when the aperture is positioned over metallic regions of the bowtie antenna [1–3]. By raster scanning the antenna over the probe at selected points in time, we obtained antenna current maps in the xy-plane [1]. The sample-probe separation was determined and controlled using automated translation stages and the amplitude and frequency dependence of the antenna resonance [1].

The THz-TDS setup was modified to include an optical pump path (Fig S1a.). The antenna with a nanowire, fabricated on a quartz substrate, was photoexcited by an optical pump pulse centered at a wavelength of 800 nm, with a fluence of 20 μJ/cm^2 and 50 μm diameter spot size. The pump

beam was incident from the transparent substrate side at a 45-degree incidence angle, with a fixed pump-probe delay of approximately 0.5 ns (i.e. exciting the sample before the THz probe pulse).

We selected a pump-probe time delay of 0.5 ns to balance two key factors: minimizing the influence of the optical pump pulse on the aperture probe and ensuring measurements are within the lifetime of charge carriers in the InAs nanowires (~500 ps) [4]. Although the signal from these charge carriers would be greater at shorter time delays, photoexcited charge carriers within the GaAs region of the aperture probe, with the lifetime of ~300 ps, would have a greater influence on the measurements. Therefore a ~0.5 ns pump-probe time delay is a practical compromise – to reduce the probe-related influences on the signal while ensuring that the charge carriers in the nanowire are still detectable.

The aperture-type near-field probe measured the transmitted THz electric field: $E(t)$ without optical excitation, and $E(t) - \Delta E(t)$ with photoexcitation. To increase the measurement sensitivity, we modulated the optical pump beam at $f_1 = 1.7$ kHz and the THz probe beam at $f_2 = 1.6$ kHz, and then demodulated the near-field probe signal at the sum frequency ($f_1 + f_2$) to extract subtle changes in the THz field, $\Delta E(t)$, and at f_2 to extract the near-field THz waveform without optical excitation, $E(t)$.

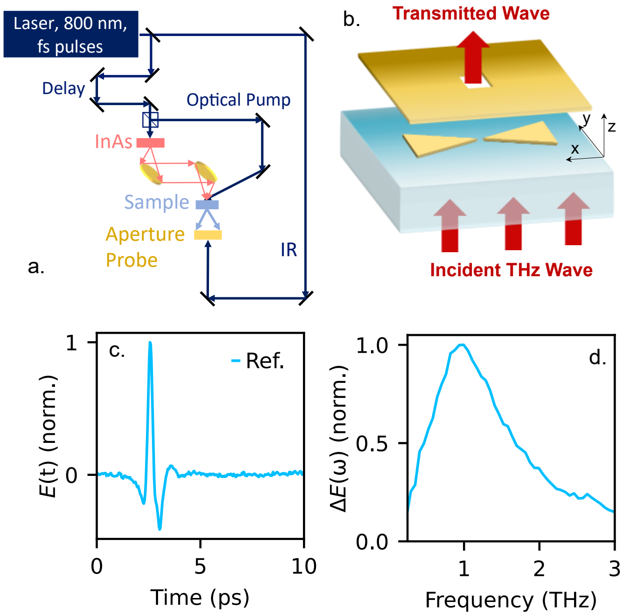

Figure S1: (a) Schematic of resonance-amplified THz near-field spectroscopy with an optical pump pulse and (b) resonant bowtie antenna with substrate mount (blue) and gold aperture probe. The bowtie antennas used in Fig. 2 were fabricated on a 0.5 mm thick GaAs substrate and are 69 μm in length, and 8 μm in width, featuring either a 5 μm open gap or $5 \times 2\ \mu m^2$ shorting metallic bar. The bowtie antennas used in Fig 3,4,5 were fabricated on a 0.5 mm thick quartz substrate and are 129 μm in length and 16 μm in width, featuring either a 5 μm open gap or a 145 nm diameter InAs nanowire. (c) Measured reference time-domain waveform acquired through a substrate-only region of the sample and (d) corresponding spectral amplitude.

2. Numerical Simulations

For the THz near-field signal modelling and quantitative evaluation of the photoexcited electron densities discussed in the manuscript, we employed a finite integration technique (FIT) full-wave electromagnetic simulations in CST Microwave Solver. This model included both the bowtie antenna with nanowire (and substrate) and the entire near-field aperture probe. The probe consisted

of a 0.3 μm thick planar gold layer with a 10 × 10 μm² aperture on a 0.5 μm thick GaAs layer, positioned 12 μm above the sample surface. The model also included a THz detector antenna, which was aligned with the aperture on the opposite side of the GaAs layer. The THz pulse was modelled as a plane wave source at normal incidence, with a shape closely matching the experimental THz pulse waveform. To simulate the near-field signal, a waveform of induced voltage was recorded between the THz detector antenna tips for each position of the bowtie antenna with respect to the probe.

To model the photoexcited response of the nanowire, we simulated a range of nanowire conductivities by varying the plasma frequency, ν, as defined by the Drude model [4]. We used a baseline ν = 6 THz, corresponding to the background charge-carrier density and a scattering rate $\gamma = 1.4 \times 10^{13}\ s^{-1}$, obtained from previous far-field studies [5]. The plasma frequency was then gradually increased, simulating the near-field signal detected by the probe at each increment. By modelling the changes in the THz electric field, $\Delta E(t)$, for different plasma frequencies, we replicated the experimentally measured pump-induced change in the THz electric field, $\Delta E(\omega)/E(\omega)$, and extracted the corresponding photoexcited charge-carrier density in the nanowire.

3. Optical Pump Pulse's Effect on Aperture-type Probe

As discussed in the manuscript, we observed a pump-induced change in both the antenna with nanowire and the reference open antenna. Since the optical pulse wavelength is centered at 800 nm, excited charge carriers within the probe's aperture region partially screen the THz field coupling through the aperture into the probe. To isolate the photoinduced response of the nanowire from that of the aperture probe, the antenna was positioned behind the center of one antenna arm, and the polarization of the optical pump pulse was varied (see manuscript Fig. 4).

4. Fabrication of Antennas with Nanowires

Nanowire Growth and Passivation: Wurtzite InAs nanowires with an average diameter of 145 ± 10 nm were grown by metal–organic chemical vapor deposition using Au nanoparticles as catalyst under the conditions described in Ref. [6]. The average nanowire diameter was measured by AFM. The nanowires were coated with ~10 nm Al_2O_3 while still on the growth substrate, using 100 cycles of trimethylaluminum and H_2O precursors at 120 °C in a Cambridge NanoTech atomic layer deposition system.

Device substrate preparation: A grid of machine-readable fiducial markers (LithoTags described in Ref. [7]), were deposited on 0.5 mm thick z-cut quartz substrates. The substrates were coated in AZ5214E photoresist and patterned using a LW-405B+, Microtech Srl Laser Writer. The markers were deposited by electron beam evaporation with Ti/Au (5/25 nm).

Nanowire transfer: The Al_2O_3-coated nanowires were then removed from the growth substrate by sonication in isopropyl alcohol and subsequently drop-cast onto the patterned substrate. The samples were dried in air at room temperature and rinsed in isopropyl alcohol to remove residue.

Fabrication of antennas with nanowires: The nanowires were then imaged under an optical microscope with a 50× objective and identified through image segmentation, with their position on the chip determined using the LithoTags. From this position database, a CAD file with THz antennas aligned to individual nanowires were generated with methods described in Ref. [7]. The antenna patterns were exposed using a LW-405B+, Microtech Srl Laser Writer system. The exposed contact regions of the nanowires were first etched in phosphoric acid to selectively remove the Al_2O_3 from the ends of the nanowire, then etched in a 2% ammonium sulfide solution to remove

the native oxide. The antennas were deposited by electron beam evaporation with Ti/Au (5nm/150nm).

REFERENCES:

1. S. Norman, J. Seddon, Y. Lu, L. Hale, A. Zaman, S. J. Addamane, I. Brener, R. Degl'Innocenti, and O. Mitrofanov, "Non-contact imaging of terahertz surface currents with aperture-type near-field microscopy," Opt. Express **32**, 24200 (2024).

2. L. L. Hale, J. Keller, T. Siday, R. I. Hermans, J. Haase, J. L. Reno, I. Brener, G. Scalari, J. Faist, and O. Mitrofanov, "Noninvasive near-field spectroscopy of single subwavelength complementary resonators," Laser Photon. Rev. **14**, 1900254 (2020).

3. I. Khromova, M. Navarro-Cía, I. Brener, J. L. Reno, A. Ponomarev, and O. Mitrofanov, "Dipolar resonances in conductive carbon micro-fibers probed by near-field terahertz spectroscopy," Appl. Phys. Lett. **107**, 021102 (2015).

4. H. J. Joyce, J. L. Boland, C. L. Davies, S. A. Baig, and M. B. Johnston, "A review of the electrical properties of semiconductor nanowires: insights gained from terahertz conductivity spectroscopy," Semicond. Sci. Technol. **31**, 103003 (2016).

5. H. J. Joyce, C. J. Docherty, Q. Gao, H. H. Tan, C. Jagadish, J. Lloyd-Hughes, L. M. Herz, and M. B. Johnston, "Electronic properties of GaAs, InAs and InP nanowires studied by terahertz spectroscopy," Nanotechnology **24**, 214006 (2013).

6. H. J. Joyce, J. Wong-Leung, Q. Gao, H. H. Tan, and C. Jagadish, "Phase perfection in zinc Blende and Wurtzite III-V nanowires using basic growth parameters," Nano Lett. **10**, 908–915 (2010).

7. T. Potočnik, P. J. Christopher, R. Mouthaan, T. Albrow-Owen, O. J. Burton, C. Jagadish, H. H. Tan, T. D. Wilkinson, S. Hofmann, H. J. Joyce, and J. A. Alexander-Webber, "Automated computer vision-enabled manufacturing of nanowire devices," ACS Nano **16**, 18009–18017 (2022).
